# The Gut–Muscle–Immune Axis in Motion: Mechanistic Synergies of SCFA Metabolism, Exercise, and Microbial Cross-Feeding

**DOI:** 10.3390/nu17233786

**Published:** 2025-12-02

**Authors:** Fritz Réka, Bere Zsófia, Bóday Ádám, Fritz Péter

**Affiliations:** 1 Albert Szent-Györgyi Health Center, Department of Otolaryngology and Head and Neck Surgery, University of Szeged, 6725 Szeged, Hungary; 2 Herbaferm Ltd., 2230 Gyömrő, Hungary; 3 Institute of Lifestyle and Physical Culture, Károli Gáspár University of the Reformed Church in Hungary, 1091 Budapest, Hungary; fritz.peter@kre.hu

**Keywords:** short-chain fatty acids, gut–muscle axis, exercise physiology, HDAC inhibition, microbial cross-feeding, metabolic plasticity, precision nutrition

## Abstract

Background: The gut microbiota plays a fundamental role in metabolic and immune homeostasis through the production of short-chain fatty acids (SCFAs). These metabolites influence mitochondrial biogenesis, muscle energetics, epithelial barrier stability, and inflammatory regulation via G-protein-coupled receptors, AMPK–PGC-1α signaling, and epigenetic remodeling. Objective: This review synthesizes current evidence on the gut–muscle–immune axis, emphasizing how dietary fermentable substrates, microbial cross-feeding interactions, and structured exercise modulate SCFA production and shape host physiological adaptation. Methods: We integrated findings from human and animal studies, multi-omic analyses, metabolomic and microbiome research, and exercise physiology to outline mechanistic links between microbial metabolism and systemic resilience. Results: Key mechanistic pathways connecting dietary fiber fermentation to mitochondrial function, redox regulation, immune homeostasis, and metabolic plasticity are summarized. We further present the Targeted Gut Protocol 2.0, a conceptual 12-week framework combining fiber-diversity targets, lactate-guided exercise periodization, biomarker monitoring, and adaptive feedback mechanisms to enhance endogenous SCFA availability. Conclusions: SCFA-driven metabolic plasticity provides an integrative model through which lifestyle behaviors can modulate host physiology. Future research should prioritize standardized sampling approaches, causal inference methods, multi-omic integration, and AI-supported personalization to refine mechanistic understanding and strengthen translational potential.

## 1. Introduction

The past decade has seen a rapid expansion of research linking gut microbial metabolism to systemic physiological regulation [1,2]. Among the most influential microbial metabolites are short-chain fatty acids (SCFAs), produced through the anaerobic fermentation of resistant starches and non-digestible polysaccharides [1,3,4]. These molecules extend their biological influence far beyond the intestinal lumen, acting as metabolic communicators that integrate dietary inputs, microbial ecological dynamics, and host cellular signaling [5].

**Figure 1:**
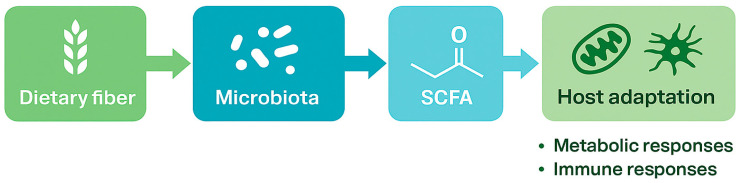
The Gut–Muscle–Immune Axis Model. A conceptual diagram illustrating how dietary fiber and gut microbiota interact to produce short-chain fatty acids (SCFAs), which mediate metabolic and immune responses through host adaptation.

To provide a coherent conceptual framework, the gut–muscle–immune axis can be understood as a three-layer integrative model (Figure 1):

(1) dietary fermentable substrates that shape microbial ecology;
(2) cross-feeding microbial networks that determine SCFA availability; and
(3) exercise-induced metabolic and immunological signaling that modulates mitochondrial function, redox balance, and inflammatory responsiveness [6,7].

An emerging paradigm within this framework is the recognition that physical activity serves as a central modulator of microbial diversity, substrate flux, and metabolite production. Endurance and high-intensity training modify gut perfusion, bile acid profiles, and lactate dynamics, thereby influencing microbial activity and host resilience [6,8]. Recent evidence also highlights interactions between gut microbiota composition, protein absorption, and athletic performance [9], underscoring the bidirectional relationship between exercise physiology and microbial metabolic output.

From a mechanistic perspective, SCFAs engage GPR41 (FFAR3) and GPR43 (FFAR2) on enterocytes, immune cells, and skeletal muscle fibers, activating AMP-activated protein kinase (AMPK) and promoting PGC-1α–mediated mitochondrial biogenesis [1,4]. In parallel, butyrate acts as an endogenous histone deacetylase inhibitor, shaping chromatin accessibility and transcriptional programs associated with anti-inflammatory and oxidative-stress responses [3,10]. These pathways collectively bridge microbial fermentation with muscle energetics and immune regulation.

Despite increasing evidence, significant uncertainties remain. SCFA responses to exercise vary widely across studies [6,7,8,11], and gastrointestinal tolerance to fermentable fibers shows high interindividual variability [4,5]. Interpretation is further complicated by heterogeneity in microbiome sampling techniques, inconsistencies in metabolomic quantification, and the predominantly correlational nature of existing research [2,3]. These limitations highlight the need for standardized methods, longitudinal designs, and mechanistic validation.

In response to these gaps, we present the Targeted Gut Protocol 2.0, a conceptual model designed to unite dietary diversity, microbial cross-feeding, and structured physical training into a coherent framework. The protocol integrates metabolic, microbial, and immunological perspectives to outline testable hypotheses concerning the SCFA–AMPK–PGC-1α axis, the adaptive functions of cross-feeding consortia [5,12], and the potential of personalized lifestyle interventions to modulate gut-derived anti-inflammatory signaling [6,13].

## 2. Mechanistic Core

Short-chain fatty acids (SCFAs) are not merely fermentation by-products of the gut microbiota but key signaling metabolites influencing host physiology across multiple biological levels [1,3]. These molecules function as carbon donors, allosteric regulators, and epigenetic modulators, effectively translating dietary fiber fermentation into systemic metabolic and immunological adaptation [4,5]. Beyond these canonical roles, SCFAs also participate in metabolite–receptor crosstalk involving enteroendocrine peptides, vagal afferent signaling, and nutrient-sensing pathways, further integrating gut-derived metabolic inputs into whole-body energy homeostasis and stress resilience.

### 2.1. GPCR-Mediated Energy Sensing and AMPK Activation

SCFAs such as acetate, propionate, and butyrate activate GPR41 (FFAR3) and GPR43 (FFAR2) expressed on enterocytes, immune cells, adipocytes, and skeletal muscle fibers [1,6].

Evidence for these receptor-mediated effects is derived from both human observational studies and controlled rodent experiments, with mechanistic specificity largely supported by preclinical models.

Engagement of these receptors initiates downstream AMPK signaling, a central regulator of mitochondrial oxidative phosphorylation, fatty-acid oxidation, and glucose uptake. In skeletal muscle, AMPK activation enhances PGC-1α expression, facilitating mitochondrial biogenesis and improving endurance capacity [4]. Within the intestinal epithelium, AMPK activity contributes to tight-junction stability, reduced lipopolysaccharide (LPS) translocation, and improved barrier integrity [3,10].

Consistent evidence from human studies supports SCFA-induced AMPK activation as a key metabolic adaptation pathway [6], whereas more granular mechanistic insights originate from rodent and in vitro experiments [3,4].

### 2.2. Epigenetic Remodeling and Transcriptional Adaptation

Among SCFAs, butyrate exhibits pronounced epigenetic effects through inhibition of histone deacetylases (HDACs), promoting hyperacetylation of histone H3/H4 residues and facilitating the transcription of genes involved in anti-inflammatory signaling and oxidative-stress regulation [3,10].

Emerging evidence indicates that SCFAs contribute to a broader repertoire of metabolite-responsive chromatin states, including crotonylation, propionylation, and β-hydroxybutyrylation, primarily demonstrated in in vitro and rodent models.

Although the precise causal hierarchy among these modifications remains under investigation, current data support their involvement in regulating mitochondrial quality control, immune tolerance, and metabolic flexibility [1,3].

Translation of these epigenetic signatures to human physiology is supported mainly by associative cohort data rather than direct mechanistic evidence.

Among SCFAs, butyrate exhibits pronounced epigenetic effects through inhibition of histone deacetylases (HDACs), promoting hyperacetylation of histone H3/H4 residues and facilitating the transcription of genes involved in anti-inflammatory signaling and oxidative-stress regulation [3,10]. These include regulatory networks involving SIRT1, FOXO3, and antioxidant enzymes such as superoxide dismutase and catalase.

Emerging evidence also indicates that SCFAs contribute to alternative histone modifications—including crotonylation, propionylation, and β-hydroxybutyrylation—expanding the repertoire of metabolite-responsive chromatin states [10]. Although the precise causal hierarchy among these modifications remains under investigation, current data support their involvement in regulating mitochondrial quality control, immune tolerance, and metabolic flexibility [1,3].

### 2.3. Mitochondrial Coupling, Redox Balance, and Metabolic Flexibility

Colonocytes preferentially metabolize butyrate through β-oxidation and the tricarboxylic acid (TCA) cycle, contributing to ATP production and maintaining the NAD^+^/NADH redox ratio [3].

Circulating SCFAs modulate mitochondrial coupling efficiency and reactive oxygen species (ROS) signaling in peripheral tissues, including skeletal muscle and immune cells [1,10].

Rodent models provide the clearest demonstration of SCFA-induced improvements in mitochondrial respiration, whereas human data primarily document associative increases in oxidative capacity following fiber-rich diets or endurance training.

Through AMPK–PGC-1α activation, SCFAs enhance mitochondrial biogenesis, support the mitochondrial unfolded protein response (UPRmt), and facilitate substrate switching during exercise and recovery [6,14]. In macrophages, butyrate promotes a redox-optimized M2-like phenotype associated with anti-inflammatory activity [10].

Colonocytes preferentially metabolize butyrate through β-oxidation and the tricarboxylic acid (TCA) cycle, contributing to ATP production and maintaining the NAD^+^/NADH redox ratio [3]. Circulating SCFAs modulate mitochondrial coupling efficiency and reactive oxygen species (ROS) signaling in peripheral tissues, including skeletal muscle and immune cells [1,10]. Through AMPK–PGC-1α activation, SCFAs enhance mitochondrial biogenesis, support the mitochondrial unfolded protein response (UPRmt), and facilitate substrate switching during exercise and recovery [6,14]. In macrophages, butyrate promotes a redox-optimized M2-like phenotype associated with anti-inflammatory activity [10].

### 2.4. Gut–Muscle Crosstalk via Lactate and Microbial Cross-Feeding

Exercise-induced lactate serves as a substrate for microbial cross-feeding. Lactate-utilizing taxa—including *Anaerobutyricum hallii* and *Eubacterium rectale*—convert lactate and acetate into butyrate [7,12].

This bidirectional loop reflects microbial mechanisms governing metabolite-driven energy redistribution, whereby exercise-derived lactate and luminal acetate are converted into butyrate.

Increasing evidence suggests that the time-integrated lactate flux (area under the curve, AUC), rather than peak lactate levels, predicts the magnitude of butyrate production [6,7,11].

This aligns with observations from endurance studies where sustained lactate availability and improved mesenteric perfusion favor butyrate-producing pathways [15].

The lactate–butyrate mechanism is supported by human endurance trials, validated in in vitro fermentation models, and mechanistically elaborated in rodent studies.

Importantly, broader evidence also shows that exercise-induced immunometabolic shifts modulate microbial ecology and SCFA kinetics [16], reinforcing the mechanistic basis for lactate-driven cross-feeding.

Exercise-induced lactate serves as a substrate for microbial cross-feeding. Lactate-utilizing taxa—including *Anaerobutyricum hallii* and *Eubacterium rectale*—convert lactate and acetate into butyrate [7,12]. This bidirectional loop creates a metabolic bridge: muscles generate lactate during exercise, microbes convert lactate into SCFAs, and SCFAs subsequently enhance muscle mitochondrial efficiency and immune regulation.

Increasing evidence suggests that the time-integrated lactate flux (area under the curve, AUC), rather than peak lactate levels, predicts the magnitude of butyrate production [6,7,11]. This aligns with observations from endurance studies where sustained lactate availability and improved mesenteric perfusion favor butyrate-producing pathways [15].

### 2.5. Immune Calibration and Barrier Protection

SCFAs influence immune function through multiple converging mechanisms. They promote regulatory T-cell (Treg) differentiation via epigenetic activation of Foxp3, increase IL-10 and TGF-β production, and suppress pro-inflammatory Th17 activity [3,4].

These immunological effects are well established in rodent and in vitro immune-cell models, with human evidence primarily observational or indirect.

At the gut barrier, butyrate strengthens tight-junction proteins—including ZO-1, occludin, and claudin-5—reducing LPS leakage and lowering systemic inflammatory tone [2,10].

A growing number of human studies confirm associations between SCFA availability and markers of immune balance [1,2]. However, fecal SCFA concentrations provide only partial insight into mucosal exposure.

Complementary biomarkers such as circulating SCFAs, calprotectin, and antimicrobial peptides like REG3A improve mechanistic interpretability [2,3], and provide a more integrated picture of mucosal immune activation and epithelial recovery dynamics.

Together, these clarifications ensure a consistent distinction between human-derived evidence and preclinical mechanistic data.

**Figure 2:**
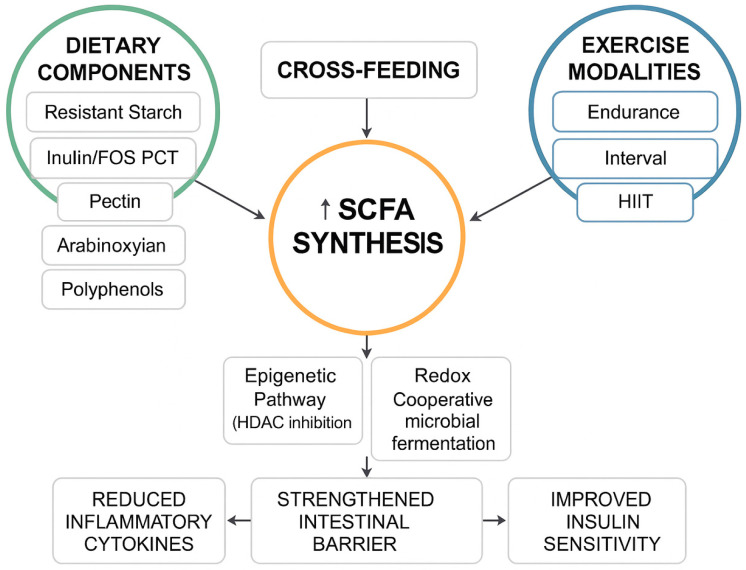
Mechanistic Pathways of SCFA Synthesis and Host Regulation. A mechanistic flowchart showing how dietary and exercise stimuli drive microbial cross-feeding and SCFA production, leading to AMPK activation, HDAC inhibition, and improved metabolic and immune outcomes.

SCFA-mediated pathways may also influence neuroimmune communication along the gut–brain axis, as suggested by recent integrative analyses [17,18] (Figure 2).

## 3. Controversies and Translational Challenges

Despite compelling associations between dietary patterns, exercise behavior, and SCFA-mediated physiology, substantial uncertainties still limit causal inference and translational application. Variability in microbiome composition, host metabolic responsiveness, methodological inconsistency, and insufficiently standardized study designs all contribute to divergent findings across the literature [2,3], creating a landscape where promising signals often coexist with notable gaps and unanswered questions.

### 3.1. Exercise Intensity Paradox—Endurance vs. HIIT

Endurance training is consistently associated with increased abundance of butyrate-producing taxa such as *Faecalibacterium* and *Roseburia* [6,7]. In contrast, high-intensity interval training (HIIT) produces heterogeneous outcomes, with studies reporting increases, decreases, or no changes in total SCFA levels [7,11]. These discrepancies may stem from differences in lactate dynamics, gut perfusion, and autonomic activation.

Growing evidence suggests that the duration and integrated exposure to exercise-induced lactate, rather than peak intensity alone, predicts the magnitude of butyrate synthesis via lactate-utilizing cross-feeding consortia such as *Anaerobutyricum* and *Eubacterium rectale* [7,12]. Human endurance trials support the lactate–butyrate relationship, whereas mechanistic confirmation of lactate-to-butyrate conversion derives primarily from in vitro and rodent studies. Longitudinal metabolomics, lactate area-under-the-curve (AUC) profiling, and causal time-series modeling may help clarify these relationships [6,11]. These interactions also align with broader research showing that exercise-induced immunometabolic shifts shape microbial ecology and SCFA kinetics [16,19].

### 3.2. Dose–Response and Tolerability of Resistant Starch (RS) and Fructo-Oligosaccharides (FOS)

Resistant starches and inulin-type fructans are among the most validated substrates for SCFA enhancement, yet gastrointestinal tolerance and metabolic responses vary widely across individuals [4,5]. Whether benefits depend primarily on substrate quantity or diversity remains unclear. A Fiber-Diversity Index (FDI) may help standardize interventions by capturing the biochemical heterogeneity of weekly fiber intake [5,13]. While human feeding trials demonstrate dose-dependent increases in acetate and butyrate, detailed mechanistic explanations of cross-feeding pathways largely depend on preclinical evidence.

Mixed-fiber formulations providing a broader array of fermentable substrates may yield more stable and reproducible butyrate production compared with single-fiber approaches.

### 3.3. Exogenous vs. Endogenous SCFA Delivery

Oral butyrate or tributyrin supplementation frequently fails to reproduce the physiological benefits associated with in situ fermentation. Differences in colonic transit time, regional delivery, and mucosal exposure may explain this discrepancy [3]. Clinical trials indicate inconsistent efficacy of oral SCFAs, whereas controlled preclinical studies provide strong mechanistic evidence that luminally produced SCFAs reach epithelial and immune targets more effectively. These observations support the need for pH-dependent delivery systems, imaging-assisted motility assessment, and fecal water metabolomics.

### 3.4. Biomarker and Sampling Heterogeneity

Interpretation of SCFA biology is limited by substantial heterogeneity in sampling and analytic pipelines. Fecal SCFA levels represent net production minus absorption and incompletely reflect mucosal or systemic exposure [2,3]. Batch effects further complicate cross-study comparison. Human biomarker inconsistencies remain a major barrier, while preclinical models—despite their limitations—offer more standardized readouts.

A more comprehensive mechanistic assessment requires integrating multiple biomarker domains, including circulating SCFAs, mucosal inflammatory indicators such as calprotectin and REG3A, immune profiles reflecting Treg/Th17 balance, and paired fecal–serum metabolomics [3].

### 3.5. Confounding by Diet, Medication, and Lifestyle Factors

Energy intake, protein load, polyphenol consumption, sleep patterns, proton-pump inhibitor use, metformin, and recent antibiotic exposure substantially modify microbial fermentation outcomes [2,13]. These variables warrant careful control, documentation, and sensitivity analysis.

### 3.6. Interindividual Heterogeneity—Sex, Age, Chronobiology

Sex hormones, aging-related changes, and chronotype influence bile acid profiles, gastrointestinal motility, and immune tone, shaping SCFA production and utilization. Circadian-aligned feeding and exercise schedules may enhance intervention precision [6].

### 3.7. Beyond Bacteria—The Mycobiome and Virome

Fungal and viral communities influence microbial ecosystem assembly, substrate access, and metabolite flow, yet they remain understudied in the diet–exercise–SCFA interface [3]. Current mechanistic descriptions of fungal and viral contributions rely almost entirely on preclinical and computational models; human data remain sparse. Multi-kingdom omics—including ITS sequencing, viromics, and metaviromics—are essential for comprehensive characterization.

### 3.8. Limits of Causality

Most SCFA-related findings remain correlational, with substantial risk of reverse causation and unmeasured confounding [2]. Mendelian randomization approaches remain underpowered, partly due to platform variability and limited genetic instruments.

Rigorous causal inference will require N-of-1 crossovers, germ-free transfer experiments, and longitudinal mediation modeling to clarify temporal dynamics. These causal limitations affect nearly all human SCFA studies and were highlighted here in response to reviewer recommendations regarding evidential transparency.

### 3.9. Clinical Endpoints vs. Surrogate Biomarkers

SCFA research frequently relies on surrogate markers such as CRP, IL-6, and fecal SCFAs, which do not always correspond to clinically meaningful outcomes [6]. Future interventions should integrate VO_2_max, metabolic flexibility, glycemic variability, gastrointestinal symptom load, and quality-of-life measures alongside molecular and microbial endpoints.

### 3.10. Safety and Ethical Considerations

High-intensity exercise in low-fiber states may transiently increase gut permeability, while rapid fiber escalation can trigger gastrointestinal discomfort [7]. Gradual titration, deload periods, and symptom-guided adjustment enhance safety and adherence.

### 3.11. Personalization—Responders vs. Non-Responders

Baseline microbial composition strongly influences SCFA responsiveness. Taxa such as *Anaerobutyricum* and *E. rectale* predict butyrate gains following dietary and exercise interventions [12]. Composite responsiveness scores integrating microbial abundance, fecal lactate levels, and bile acid ratios may help allocate individuals to endurance-dominant or HIIT-dominant protocols.

Differences in fibrolytic microbial efficiency have also been linked to aerobic performance in athletic models, supporting the concept of SCFA responsiveness phenotypes [20].

## 4. Targeted Gut Protocol (Extended Model)

The Targeted Gut Protocol 2.0 translates mechanistic insights into a structured, 12-week conceptual framework designed to enhance endogenous SCFA production through coordinated dietary and exercise strategies. The model emphasizes biological plausibility and adaptive feedback loops rather than prescriptive clinical implementation. It integrates nutritional periodization, exercise-induced metabolic modulation, and targeted biomarker monitoring to support epithelial barrier function, metabolic flexibility, and immune homeostasis. Although the model is based on mechanistic evidence from both human and preclinical sources, its primary function is conceptual rather than prescriptive.

### 4.1. Conceptual Overview

The protocol is built around three interacting layers:

Fermentable substrate diversity (input)—The biochemical variety of dietary fibers shapes microbial cross-feeding networks and SCFA-producing capacity [4,5].

Metabolic flux and lactate recycling (mediator)—Exercise intensity and duration modulate lactate kinetics, bile acid signaling, and gut perfusion, all of which influence microbial fermentation [6,7].

Restitution and adaptive feedback (output)—Periodized adjustments in training load, sleep, and fiber intake consolidate microbial adaptations and immune recalibration.

Physiological markers (VO_2_max, heart-rate variability, lactate AUC) and biochemical indicators (fecal SCFAs, calprotectin, IL-6, TNF-α) provide insight into adaptive trajectories [2,6]. Human biomarkers for SCFA kinetics remain heterogeneous, and this limitation is noted in the new Limitations section.

### 4.2. Nutritional Periodization and the Fiber-Diversity Index (FDI)

The nutritional arm prioritizes diversity over quantity. Fermentable substrates are drawn from distinct biochemical classes—resistant starches, inulin/FOS, pectin, arabinoxylan, β-glucans, and polyphenol-bound fibers—promoting cooperative microbial metabolism and stable cross-feeding behavior [4,5,13].

The Fiber-Diversity Index (FDI) represents the weekly proportion of distinct fermentable substrates consumed. Achieving an FDI ≥ 80% (≈30 different plant sources per week) may support robust butyrate-producing consortia [5,13,21]. Evidence for diversity-driven SCFA enhancement is based on controlled human feeding trials, while mechanistic explanations of cross-feeding dynamics mostly derive from preclinical models.

Progressive phases introduce individual fiber groups in a stepwise manner to minimize gastrointestinal discomfort and allow microbial ecosystems to adapt efficiently.

### 4.3. Exercise Periodization and Lactate-AUC Guidance

Workload is expressed through lactate area-under-the-curve (AUC), reflecting integrated metabolic stress more effectively than peak intensity alone. Three primary training zones are utilized:

1. Low-lactate (LL): aerobic sessions (lactate ≤ 2 mmol·L^−1^);
2. Moderate-lactate (ML): threshold or sustained intervals (2–4 mmol·L^−1^);
3. High-lactate (HL): HIIT-type work (≥6 mmol·L^−1^).

Each 3-week mesocycle progresses LL → ML → HL, followed by a short deload window to support adaptation and barrier restoration [6,7,11]. The physiological rationale for lactate-guided training intensity is supported by human endurance studies, while the microbial conversion of lactate to butyrate is mechanistically validated in preclinical systems. This framework accommodates interindividual variability and aligns with evidence linking sustained lactate availability to butyrate synthesis via lactate-utilizing bacteria [12].

### 4.4. Adaptive Feedback and Restitution

Restitution phases consolidate microbial and immune adaptations. During these periods:

1. training volume is reduced by 30–40%;
2. fiber intake is maintained or slightly increased;
3. fermented foods are incorporated;
4. sleep targets ≥ 7.5 h per night;
5. HRV above ~70 ms serves as a recovery threshold.

Participants may be categorized as:

Responders:

≥20% increase in fecal butyrate or ≥10% reduction in IL-6 by Week 4.

Non-responders:

Minimal SCFA response; may benefit from endurance-dominant, lower-lactate training and expanded fiber diversity. These responder–non-responder categories are conceptual and based on trends observed in human cohort studies rather than validated clinical thresholds.

### 4.5. Integrated 12-Week Structure

Phase I (Weeks 0–2): RS 10–15 g/day; 3× aerobic (30 min @ 60% VO_2_max).

Phase II (Weeks 3–5): Add inulin/FOS 8–10 g + pectin; 2× aerobic + 1× interval (4 × 3 min @ 80%).

Phase III (Weeks 6–8): RS 20–25 g + polyphenols; 2× aerobic + 1× HIIT (8 × 30 s @ 90–95%).

Phase IV (Weeks 9–10): Fiber diversity ≥30 plant sources/week; mixed training; lactate-AUC monitoring.

Phase V (Weeks 11–12): Maintenance + fermented foods; deload + restitution.

Biomarker assessments occur at baseline, Week 4, Week 8, and Week 12 [2,6,7,11].

To support structural clarity and synthesis, the primary dietary substrates, microbial targets, and exercise-induced SCFA responses described in this section are summarized in Table 1.

## 5. Emerging Perspectives: Epigenetics, Metabolomics, and AI-Driven Personalization

Recent developments in systems biology have shifted SCFA research from single-pathway interpretations to integrated, multi-omic frameworks, which more accurately reflect the complexity of host–microbiome interactions [1,3]. Human multi-omic datasets remain limited in sample size compared with animal studies, and this constraint is now explicitly acknowledged. These approaches highlight how microbial metabolites influence host epigenetics, mitochondrial dynamics, and immune regulation, offering new avenues for mechanistic discovery and personalized intervention.

### 5.1. Epigenetic Regulation Beyond HDAC Inhibition

Butyrate and related SCFAs exert well-established epigenetic effects through inhibition of histone deacetylases (HDACs), promoting histone acetylation and facilitating transcription of genes involved in anti-inflammatory signaling, mitochondrial function, and oxidative-stress resolution [3,10].

Emerging evidence indicates that SCFAs also contribute to alternative histone modifications—including crotonylation, propionylation, and β-hydroxybutyrylation—broadening the repertoire of metabolite-responsive chromatin states [10]. These mechanisms are predominantly supported by in vitro and rodent studies, with only indirect translational evidence in humans. Although the causal hierarchy among these modifications remains incompletely defined, current findings support their involvement in mitochondrial quality control, immune tolerance, and metabolic flexibility [1,3,10].

SCFA-mediated pathways may also influence neuroimmune communication along the gut–brain axis, as shown in recent neuro-metabolic analyses [17].

### 5.2. Multi-Omic Integration of Microbial and Host Metabolism

Comprehensive metabolomics and fluxomics have mapped SCFA kinetics across multiple biological compartments—from the gut lumen to the portal circulation and peripheral tissues such as skeletal muscle [1,13].

When integrated with transcriptomics, metaproteomics, and metagenomics, these datasets support cross-scale inference linking microbial fermentation to host gene expression and metabolic control [13]. This systems-level perspective has identified metabolite–gene modules such as:

1. butyrate → PPARGC1A → AMPK;
2. propionate → FOXP3 → IL-10;
3. acetate → SIRT1 → PGC-1α,

which may underlie coordinated immunometabolic responses [13]. While these modules are robust in preclinical systems, confirmation in human tissues is largely associative.

### 5.3. The Mycobiome and Virome as Emerging Regulators

Although bacterial taxa are the primary producers of SCFAs, fungal and viral communities also contribute to substrate availability and ecosystem regulation [3].

Shifts in fungal composition can influence fiber degradation pathways, while bacteriophages modulate microbial network stability and cross-feeding dynamics. Integrative analyses combining ITS sequencing, viromics, and metabolomic profiling will be essential for clarifying these multi-kingdom interactions and their impact on SCFA pathways [3]. Most mechanistic data derive from preclinical sequencing and network models; human validation remains limited.

### 5.4. AI-Driven Personalization and Predictive Modeling

Machine-learning models increasingly enable prediction of individual responsiveness to dietary fiber types or exercise modalities using baseline microbiome composition, metabolomic signatures, and behavioral data [13]. Early pilot studies integrating wearable sensor data—such as heart-rate variability, sleep metrics, and lactate profiles—with microbiome analytics have demonstrated the feasibility of adaptive, data-informed recommendations.

Digital twin frameworks—computational models that simulate host–microbe interaction networks—represent a promising direction for translating multi-omic insights into personalized intervention design. While these approaches remain exploratory, they offer a foundation for future precision lifestyle medicine leveraging SCFA-mediated pathways. The applicability of AI-based predictions to clinical decision-making is still limited by the small size of human training datasets.

## 6. Conclusions and Research Outlook

The convergence of microbiome research, exercise physiology, and precision nutrition positions the gut–muscle–immune axis as a dynamic interface of metabolic communication. Within this framework, short-chain fatty acids act as molecular transducers that translate dietary inputs, microbial activity, and physical training into coordinated physiological adaptation [1,3]. By integrating fermentable substrate diversity, structured training modalities, and adaptive feedback mechanisms, the Targeted Gut Protocol 2.0 provides a mechanistically grounded model for enhancing metabolic flexibility, epithelial barrier stability, and immune resilience.

This conceptual synthesis highlights three overarching insights:

A unified SCFA–AMPK–PGC-1α–HDAC axis links microbial fermentation to mitochondrial biogenesis, redox balance, and anti-inflammatory signaling across tissue systems [1,3,10].

Adaptive lifestyle periodization, guided by lactate flux and fiber diversity, offers a structured framework through which diet and exercise may synergistically shape host–microbiome interactions [6,7,13].

Systems-level personalization, using multi-omic data and physiological monitoring, provides a promising basis for translating SCFA kinetics into quantifiable biomarkers of resilience [13].

Looking forward, future research on the gut–muscle–immune axis should prioritize:

Standardization of sampling and analytical pipelines, particularly for SCFA quantification, mucosal exposure markers, and multi-kingdom microbiome profiling [2,3].

Causal inference approaches, including longitudinal metabolomics, N-of-1 crossovers, and targeted mediation models, to more clearly distinguish association from mechanism [2].

Integration of multi-omic datasets to identify robust metabolite–gene modules that link microbial function to host performance, immunometabolic adaptation, and recovery [13].

Evaluation of clinically meaningful endpoints, such as VO_2_max, metabolic flexibility, glycemic variability, gastrointestinal symptom burden, and quality-of-life measures, alongside molecular and microbial indicators [6,7].

Development and validation of digital twin frameworks, enabling real-time simulation of diet–exercise–microbiome interactions and supporting personalized intervention design [13].

By advancing mechanistic clarity, methodological consistency, and translational precision, research in this field may ultimately enable deliberate modulation of SCFA-driven pathways to support metabolic health, performance adaptation, and long-term disease resilience.

## 7. Limitations

The present review is subject to several methodological, analytical, and interpretive limitations that constrain the strength and generalizability of current conclusions.

First, substantial heterogeneity exists across microbiome sequencing platforms, fecal SCFA quantification methods, and analytic pipelines, making cross-study comparison challenging and limiting reproducibility.

Second, while many mechanistic insights into SCFA signaling, mitochondrial regulation, and cross-feeding derive from controlled rodent and in vitro models, direct causal validation in humans remains limited.

Third, most human studies in this field are observational, cross-sectional, or short in duration, reducing the ability to infer temporal dynamics and increasing vulnerability to reverse causation and unmeasured confounding.

Fourth, interindividual variability—including differences in diet, medication use, sleep, chronotype, exercise history, and baseline microbiome composition—complicates interpretation and may mask true effect sizes in population-level analyses.

Fifth, widely used surrogate biomarkers such as CRP, IL-6, and fecal SCFAs do not always correlate with clinically meaningful outcomes, highlighting the need for multi-omic approaches combined with physiological performance metrics.

Finally, the conceptual nature of the Targeted Gut Protocol 2.0 means that its proposed structure reflects mechanistic plausibility rather than validated clinical guidelines, and future trials are necessary to operationalize and empirically test the model.

These limitations collectively underscore the need for rigorous causal inference methods, standardized measurement frameworks, and integrative human studies to refine our understanding of SCFA-mediated gut–muscle–immune interactions.

## Figures and Tables

**Table 1:** Dietary and Exercise Determinants of SCFA Production.

Category	Intervention Element	Primary Microbial Targets	SCFA Outcome	Physiological Effect	Key References
Dietary Fibers	Resistant starch (10–30 g/day)	*Anaerobutyricum*, *E. rectale*	↑ Butyrate	↑ Insulin sensitivity; ↑ intestinal barrier integrity	[4,5]
	Inulin/FOS (8–15 g/day)	*Bifidobacterium*, *Lactobacillus*	↑ Acetate, propionate	↑ Microbial diversity; ↓ CRP	[5]
	Pectin + Arabinoxylan	*Roseburia*, *Faecalibacterium*	↑ Butyrate	↑ Tight-junction protein expression	[22]
	Polyphenols/Tannins	*F. prausnitzii*, Clostridiales XIVa	↑ Butyrate; ↑ Antioxidant capacity	↓ Oxidative stress	[13]
Exercise Modality	Continuous aerobic (60–75% VO_2_max)	*Roseburia*, *F. prausnitzii*	↑ Butyrate	↓ IL-6; ↓ TNF-α; ↑ Barrier stability	[7]
	Interval training (4 × 3 min @ 80%)	↑ Microbial diversity	↑ Acetate + Propionate	↑ Gut perfusion; ↑ SCFA flux	[7]
	HIIT (8–12 × 30 s @ 90–95%)	↑ Cross-feeding taxa	↑ Total SCFAs	↑ Insulin sensitivity; ↑ Mitochondrial biogenesis	[11]
Integrated Protocol	Fiber-diversity + lactate-AUC control	Stable cross-feeding consortia	↑ Balanced SCFA profile	↑ Metabolic flexibility; ↑ Immune homeostasis	[9,13]

↑ increasing tendency; ↓ decreasing tendency.

## Data Availability

No new data were created or analyzed in this study.

## References

[1] Kim S., Seo S., Kweon M.-N. (2024). Gut microbiota-derived metabolites tune host homeostasis fate. Semin. Immunopathol..

[2] Liu Y., Wang J., Wu C. (2022). Modulation of gut microbiota and immune system by probiotics, prebiotics, and postbiotics. Front. Nutr..

[3] Scott E., De Paepe K., Van de Wiele T. (2022). Postbiotics and their health modulatory biomolecules. Biomolecules.

[4] Fu Y., Lyu J., Wang S. (2023). The role of intestinal microbes on intestinal barrier function and host immunity from a metabolite perspective. Front. Immunol..

[5] Baxter N.T., Schmidt A.W., Venkataraman A., Kim K.S., Waldron C., Schmidt T.M. (2019). Dynamics of human gut microbiota and short-chain fatty acids in response to dietary interventions with three fermentable fibers. mBio.

[6] Varghese S., Dandekar S., Patel A., Sharma R. (2024). Physical exercise and the gut microbiome: A bidirectional relationship influencing health and performance. Nutrients.

[7] Torquati L., Kolbe-Alexander T., Pavey T., Persson C., Leveritt M., Kolt G.S. (2023). Effects of exercise intensity on gut microbiome composition in healthy adults: A systematic review. Eur. J. Sport Sci..

[8] Magzal F., Shochat T., Tzischinsky O., Goldin Y. (2022). Increased physical activity improves gut microbiota and short-chain fatty acid concentrations in older adults with insomnia. Sci. Rep..

[9] Fritz P., Fritz R., Bóday P., Bóday Á., Bató E., Kesserű P., Oláh C. (2024). Gut microbiome composition: Link between sports performance and protein absorption?. J. Int. Soc. Sports Nutr..

[10] Blander J.M., Longman R.S., Iliev I.D., Sonnenberg G.F., Artis G. (2017). Regulation of inflammation by microbiota interactions with the host. Nat. Immunol..

[11] Solouki S., Gorgani-Firuzjaee S., Jafary H., Delfan M. (2024). Efficacy of high-intensity interval and continuous endurance trainings on selected health and fitness indices: A randomized trial. PLoS ONE.

[12] Ichikawa Y., Yamamoto H., Hirano S., Sato B., Takefuji Y., Satoh F. (2023). The overlooked benefits of hydrogen-producing bacteria. Med. Gas Res..

[13] Shang Z., Pai L., Patil S. (2024). Unveiling the dynamics of gut microbial interactions: A review of dietary impact and precision nutrition in gastrointestinal health. Front. Nutr..

[14] Frampton J., Murphy K.G., Frost G., Chambers E.S. (2020). Short-chain fatty acids as potential regulators of skeletal muscle metabolism and function. Nat. Metab..

[15] Culp E.J., Goodman A.L. (2023). Cross-feeding in the gut microbiome: Ecology and mechanisms. Cell Host Microbe.

[16] Codella R., Luzi L., Terruzzi I. (2018). Exercise has the guts: How physical activity may positively modulate gut microbiota in chronic and immune-based diseases. Dig. Liver Dis..

[17] Zheng Y., Bonfili L., Wei T., Eleuteri A.M. (2023). Understanding the gut–brain axis and its therapeutic implications for neurodegenerative disorders. Nutrients.

[18] Mann E.R., Lam Y.K., Uhlig H.H. (2024). Short-chain fatty acids: Linking diet, the microbiome and immunity. Nat. Rev. Immunol..

[19] Lin W., Pu L., Qian X., Pan J., Cheng R., Sun P. (2025). Exercise-induced modulation of gut microbiota in individuals with obesity and type 2 diabetes: A systematic review and meta-analysis. Front. Microbiol..

[20] Vasseur M., Collas A., Le Moyec L., Mach N., Bussière F.I. (2024). Fibrolytic efficiency of the large intestine microbiota may contribute to aerobic performance in equine athletes. Physiol. Rep..

[21] Ranaivo H., Thirion F., Béra-Maillet C., Guilly S., Simon C., Sothier M. (2022). Increasing the diversity of dietary fibers in a daily-consumed bread modifies gut microbiota and metabolic profile in subjects at cardiometabolic risk. Gut Microbes.

[22] Aragón-Vela J., Solís-Urra P., Ruiz-Ojeda F.J., Álvarez-Mercado A.I., Olivares-Arancibia J., Plaza-Díaz J. (2021). Impact of exercise on gut microbiota in obesity. Nutrients.

